# Automatic temporal analysis of speech: A quick and objective pipeline for the assessment of overt stuttering

**DOI:** 10.3758/s13428-025-02733-z

**Published:** 2025-07-18

**Authors:** Vishruta Yawatkar, Ho Ming Chow, Evan Usler

**Affiliations:** 1 Center for Bioinformatics and Computational Biology, University of Delaware, 590 Avenue 1743, Suite 147, Newark, DE 19713 USA; 2 Department of Communication Sciences and Disorders, University of Delaware, 100 Discovery Blvd, Newark, DE 19713 USA

**Keywords:** Stuttering, Pausing, Speech production, Speech motor control, Fluency

## Abstract

Fluency disorders, such as developmental stuttering, have been characterized by behavior such as blocks, repetitions, and prolongations in speech. Accurate measurement of overt stuttering behavior can aid in diagnostic evaluation and the determination of optimal treatment for this disorder. This study proposes a method – Automatic Temporal Analysis of Speech (ATAS) – for the assessment of speech fluency based on the detection and quantification of discrete pauses and vocal events. Our ATAS metrics include speech rate, total pause time, pause count, mean pause duration, mean vocal duration, pause duration variability, and vocal duration variability. We used oral reading audio samples from a total of 35 English-speaking participants: 17 from adults who stutter (AWS) and 18 from adults who do not stutter (AWNS). AWS, in general, exhibited more pausing or hesitancy in speech compared to AWNS, as evidenced by slower speech rate, greater total pause time, higher pause count, and longer mean duration of pause events. Numerous pause and vocal metrics acquired from ATAS were correlated with a canonical measure of stuttering frequency percent syllables stuttered, suggesting that automatically detected temporal metrics of pause and vocal events within continuous speech are highly associated with overt stuttering behavior. ATAS metrics generally predicted the status of each participant as either an AWS or AWNS grouping with accuracies considerably higher than random guessing using random forest and LSTM classifiers. This pipeline may provide an alternative and complementary method that speech-language pathologists and other health professionals can use in the assessment of fluency disorders.

## Introduction

Developmental stuttering is a communication disorder characterized by overt stuttering behaviors, such as blocks, repetitions, and prolongations, during speech production. Accurate measurement of overt stuttering behavior can aid in diagnostic evaluation, determination of an optimal course of treatment, and for tracking progress in that treatment. However, stuttering is characteristically variable – with overt stuttering severity (e.g., intensity and frequency of stuttering behaviors), along with the environmental conditions associated with that severity, often differing across individuals. In other words, stuttering is a complex social behavior, and quantifying such behavior objectively and efficiently is not an easy matter. Overt stuttering behavior has been traditionally quantified as moments of discrete stuttering ‘disfluencies’ as perceived by an observer/listener (Bloodstein et al., 2021; Yairi & Ambrose, 2004). Over the past century, stuttering behaviors have been classified and quantified using disfluency-type typologies and taxonomies (Johnson, 1961; Yairi & Ambrose, 2004). These attempts to classify stuttering behavior have contributed to our understanding of the nature and developmental trajectory of overt stuttering behavior, particularly in children who stutter (Walsh et al., 2020).

The ability of the speech-language pathologist (SLP), or any listener, to assess stuttering severity via the subjective identification of overt stuttering behavior has long been questioned theoretically and clinically (Curlee, 1981; Perkins, 1984). First, stuttering behaviors exhibit a conceptual vagueness that hinders reliable and easy identification from other forms of speech disfluency. Despite reported ease of perceiving categorical differences between stuttered and non-stuttered speech (Hamre, 1992), considerable inter-judge *un*reliability has been reported, even among fluency specialists (Bothe, 2008; Valente et al., 2015). Differences in methods and procedures for the quantification of overt stuttering behavior have led to significant variability in assessment (Howell et al., 2011). Second, the more holistic concept of stuttering severity is also vague, as the phenomenology of the disorder includes overt and covert behaviors, cognitions, and emotions that vary considerably across individuals who stutter (Tichenor & Yaruss, 2018). The subjective judgment of stuttering can differ significantly across SLPs as a function of clinical experience (Brundage et al., 2006). In sum, Bloodstein and colleagues affirm “any measure of severity of stuttering that requires listener identification of stuttered moments is based upon an evaluative process subject to such troublesome uncontrolled variables as the standards, definitions, or criteria of the person who is doing the identifying” (p. 5) (Bloodstein & Ratner, 2007). Given the ambiguities and difficulties that appear inherent to previous methods in subjectively assessing overt stuttering behavior, alternative methods of quantifying speech fluency that are objective, instrumental, consistent, and rapid will hopefully contribute to established methodologies of stuttering assessment.

### Temporal analyses of pause and vocal events in speech

In this preliminary study, we propose a novel method for the assessment of speech fluency based on the automatic detection and quantification of discrete pauses and vocal events in continuous speech. A pause is commonly defined as a “temporary stop,” a “brief suspension of the voice,” or a “temporary inaction especially caused by uncertainty (Merriam-Webster, n.d.).” Our study interprets a “pause event” during talking in the spirit of these simple definitions – the lack of speech production. Inversely, a “vocal event” is an instance of uttered speech. However, the gestalt of speech pausing creates a structured sequence of temporal periods that enhance the rhythm and fluency of speech production (Goldman-Eisler, 1972). Speech pausing is typically brief, does not disrupt the flow of speech, and enhances communication by providing emphasis or clarity (Ratcliff et al., 2002). Continuous speech, including oral reading across various languages, has been shown to exhibit regular pausing of both short and long durations (Campione & Véronis, 2002). More specifically, pause durations during oral English reading exhibited a bimodal distribution, with the short pauses having a mean duration of ~125 ms and long pauses having a mean duration of ~500 ms. This use of silence during speech is often intentional (e.g., discourse marker) and pragmatic, such as signaling a change in topic or conveying emphasis or emotion (Atmaja & Akagi, 2020a, b; Duez, 1982). However, pausing may also be unintentional, involuntary, and/or perceived by the speaker to be outside their agentic control (McClay & Osgood, 1959). In this sense, speech pause analysis can give us a sense of the fluency (i.e., efficiency) of the speech motor and language performance.

A quantitative analysis of the temporal nature of speech, in the form of pause and vocal events, is an alternative approach for characterizing overt stuttering behavior and its frequency. The degree of pausing during oral reading appears to be more closely related to listeners’ perception of stuttering severity than more traditional and categorical measures of stuttering assessment (Prosek et al., 1979). Recent studies have implicated atypical mechanisms of speech inhibition in the etiology of stuttering (Arenas, 2017; Neef et al., 2018; Orpella et al., 2023). Atypical speech pausing has been a long considered, yet often neglected, marker of speech (dis)fluency and clinical feature of stuttering (Afroz & Koolagudi, 2019; Beltrame et al., 2011; Love & Jeffress, 1971; Rieber et al., 1972; Wingate, 1984). Pauses observed in stuttered speech are often characteristically different than in non-stuttered speech in frequency and duration (Krivokapić, 2009; Teixeira et al., 2017). Even when perceptually fluent, adults who stutter (AWS) generally produce more pauses than adults who do not stutter (AWNS) (Love & Jeffress, 1971).

Previous studies have used different semi-automatic and automatic methods to quantify temporal characteristics, such as silent pauses, of continuous speech (Afroz & Koolagudi, 2019; Green et al., 2004; Teixeira et al., 2017). A study based on a comparative analysis of speech in people with amyotrophic lateral sclerosis and a control group used a MATLAB-based Speech Pause Analysis (SPA) to detect various metrics of the frequency, duration, and variability of speech and pause events from an acoustic signal (Green et al., 2004). SPA calculated these metrics based on three threshold values: minimum pause event duration, minimum speech event duration, and minimum signal amplitude. Teixeira et al. also attempted to automatically detect pauses in spontaneous speech using an algorithm based on signal energy and the zero-crossing rate (ZCR) evaluation, which was compared to threshold values relative to “silent” sections of the audio data (Teixeira et al., 2017). Such semi-automatic methods for quantifying temporal parameters of speech have been quite powerful in distinguishing the speech of individuals with various speech disorders, including dysarthria (König et al., 2015; Tanchip et al., 2022; Toth et al., 2018). However, they do not provide a complete automated pipeline to detect silent and vocal events in the audio file, visualize them, and analyze the temporal data to obtain cumulative statistics such as the frequency and duration variables of the events along with individual event data. The data obtained from such a pipeline can be used as an input in classifiers as well as time-series models that use machine learning algorithms that can distinguish the speech of those who stutter from those who do not.

Lastly, many studies in the past have used various statistical and machine learning approaches to identify stuttering behavior in continuous speech (Barrett et al., 2022). New and improved machine learning models are being developed that use favorable acoustic metrics for the classification of various classes of fluent and disfluent speech. However, many of these models use both spectral and temporal features for model training, e.g., to classify disfluency types (Lea et al., 2021; Sheikh et al., 2022). Some of them use mainly pause events to extract features to train models to classify different types of speech (Igras-Cybulska et al., 2016).

### Purpose of the current study

The automatic and instrumental detection and quantification of pauses and vocal events during speech may provide an alternative and complementary method that SLPs and other health professionals can use in the assessment of fluency disorders, such as stuttering. Application of such a method during an oral reading task allows for an analysis of continuous speech production while controlling for the dynamic cognitive, linguistic, and motoric complexities of everyday speech. We have constructed a novel methodology, Automatic Temporal Analysis of Speech (ATAS), to quantify the temporal regularities of pause and vocal events within the speech stream.

Does the speech of AWS differ from AWNS in the frequency and duration of vocal and pause events? Can automatic quantification of these temporal parameters provide an alternative method for the quantification of overt stuttering frequency? ATAS produces a series of temporally based output metrics regarding the frequency, duration, and variability of discrete pause and vocal events, and in doing so, provides a quantitative proxy of speech (dis)fluency. In our study, AWS and AWNS participants orally read an age-appropriate passage in a single attempt, in a natural-speaking voice, and at a comfortable pace. The fluency of these productions, including the presence of overt stuttering behavior, was not altered before our analyses. ATAS speech metrics were applied to recorded audio files of these productions. These metrics were similar to those quantified by SPA (Green et al., 2004). However, unlike previous methods, ATAS did not require any manual selection of silent intervals and uses an approach that blindly sections an acoustic signal in an unsupervised manner into non-overlapping segments of equal size. It did not use any spectral input and was based on analysis only in the temporal domain for the detection of discrete vocal and pause events. Our seven ATAS metrics relevant to the current study include:

i. *Speech rate:* words spoken per minute.
ii. *Total pause time (s)*: total time spent pausing while speaking.
iii. *Pause count*: total number of pause events while speaking.
iv. *Mean pause duration (ms)*: mean duration of all pause events while speaking.
v. *Mean vocal duration (ms)*: mean duration of all vocal events while speaking.
vi. *Pause duration variability*: coefficient of variation of pause event duration while speaking.
vii. *Vocal duration variability*: coefficient of variation of vocal event duration while speaking.

We have three central hypotheses. Our first hypothesis is that AWS will exhibit more pausing or hesitancy in speech compared to AWNS, as evidenced by slower speech rate, greater total pause time, higher pause count, and longer mean duration of both pause and vocal events. These measures of pausing should be strongly correlated with stuttering frequency, as indexed by %SS. %SS should increase with greater pause count and greater total pause time. %SS should decrease with faster speech rate and longer mean duration of pause and vocal events. Our second hypothesis is that the speech of AWS will be more temporally irregular than AWNS, as evidenced by greater variability in the duration of pause and vocal events. These measures of temporal irregularity should be positively correlated with stuttering frequency, as indexed by percent syllables stuttered (%SS), a common metric of overt stuttering frequency (Karimi et al., 2014). Given pausing during English oral reading consists of a bimodal distribution of short and long pauses (Campione & Véronis, 2002), in an exploratory analysis, we will include separate analyses for short (50–150 ms) and long (>150 ms) pauses. Short pauses have a lower threshold of 50ms to reduce the influence of non-speech noise artifacts being detected as vocal events. We do not have a priori hypotheses regarding short and long pause durations. Our third hypothesis is that our ATAS metrics will be accurate in predicting the status of each participant as either an AWS or AWNS grouping. We will explore a classification model based on ATAS-generated time-series feature data and conduct an analysis based only on features calculated using ATAS metrics to train classifiers to blindly distinguish the speech of AWS from AWNS.

## Methods

### Participants

In this study, data from a total of 35 English-speaking participants were used: 17 AWS and 18 AWNS. AWS participants (mean age = 39.59 years; SD = 14.45; range = 24–62 years; 12 male and 5 female) were retrieved from the FluencyBank database (Bernstein Ratner & MacWhinney, 2018). A similar group of AWNS (mean age = 31.78 years; SD = 9.95; range = 22–63 years; 10 male and 8 female) was locally recruited. Participants were labeled as either AWS or AWNS by self-identification. All participants read the Friuli passage from the Stuttering Severity Index-Fourth Edition (SSI-4; See Appendix 1) (Riley & Bakker, 2009). This adult-level reading passage contains 236 words and 369 syllables. The participants were instructed to read the passage aloud at their habitual speech rate and loudness. A canonical method for quantifying the frequency of stuttering behavior, percent syllables stuttered (%SS), was also determined for each AWS participant from the oral reading (Bloodstein et al., 2021). %SS was rated by a graduate research assistant trained by the last author. The analysis consisted of inspecting audio-visual recordings from the ‘English Voices-AWS Corpus’, which is publicly available via FluencyBank. To establish inter-rater reliability, 1/3 of AWS recordings were also rated by a licensed SLP with clinical experience in stuttering on our research team. Regarding intra-rater agreement, all ratings by the primary rater differed by less than 1.0 %SS. Regarding inter-rater agreement, 80% of ratings by the secondary rater differed by less than 1.0 %SS and 100% differed by less than 2.0 %SS. This study protocol was approved by the University of Delaware Institutional Review Board.

### Data processing

The acoustic data from AWS and AWNS were analyzed using a custom Python-based ATAS pipeline. This pipeline involves preprocessing the audio file to perform noise reduction and trimming. A temporal and amplitude thresholding method based on the acoustic energy and a zero-crossing rate (ZCR) was used to detect the vocal and pause segments in the file. Our ATAS pipeline followed the following procedure: First, noise reduction based on spectral gating was performed to remove unnecessary ambient noise from each participant’s audio recording of their oral reading. Noise reduction involved filtering based on noise-gating frequencies using the power of the signal as compared to the noise-only period, which is detected automatically (Inouye et al., 2014). Second, each audio file was trimmed, if required, based on time point inputs. Participant files were clipped to remove extraneous audio content before the first word and after the last word of the reading passage. Trimmed audio files were then normalized by amplitude. The normalized data was then analyzed to detect vocal and pause events.

**Fig. 1:**
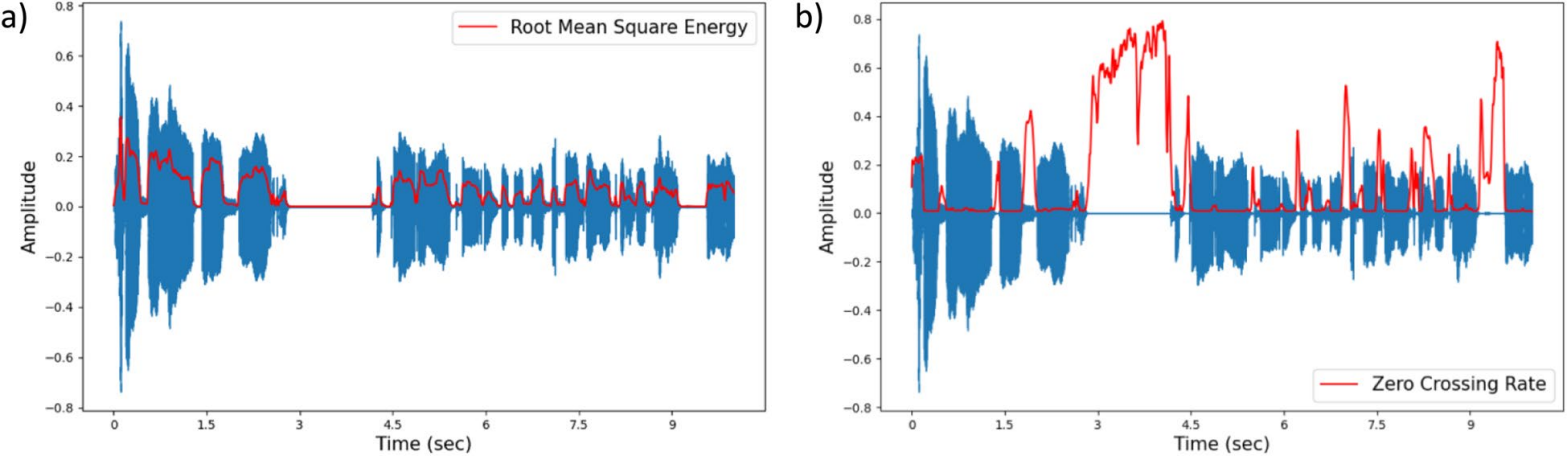
Time series plots of the same signal from a sample AWNS participant. a) Root mean square energy of the signal quantifies the energy or the loudness of the signal. **b)** Zero crossing rate is the rate at which the audio waveform crosses the zero axis over a specific time period

The ATAS algorithm is based on two parameters for setting the detection thresholds. These are the short-term root mean square energy (RMSE) and the ZCR of the noise-reduced signal. The RMSE of a signal is the square root value of the signal energy where the signal energy is calculated by summing the squares of the absolute values frame-wise. The energy of a signal corresponds to the total signal magnitude (Fig. 1a). The algebraic sign of the acoustic signal fluctuates between positive and negative. The ZCR is the total number of times the amplitude of the signal has crossed the zero value. RMSE and ZCR are defined as:

$$\begin{array}{l}{E}_{rms}=\sqrt{\frac{1}{M}\sum\limits_{m=1}^{M}\frac{1}{N}\sum\limits_{n=0}^{N-1}x{\left[m+n\right]}^{2}}\\ {E}_{zcr}=\sqrt{\frac{1}{M}\sum\limits_{m=1}^{M}\frac{1}{N}\sum\limits_{n=0}^{N-1}\left|\text{sign}\left(x\left[m+n\right]\right)-\text{sign}\left(x\left[m+n-1\right]\right)\right|}\end{array}$$

where,

*M*: is the number of subsegments analyzed,
*N*: is the number of samples per frame in each subsegment,
sign(x): is the sign function, which returns 1 for positive values, − 1 for negative values, and 0 for zero crossings.

**Fig. 2:**
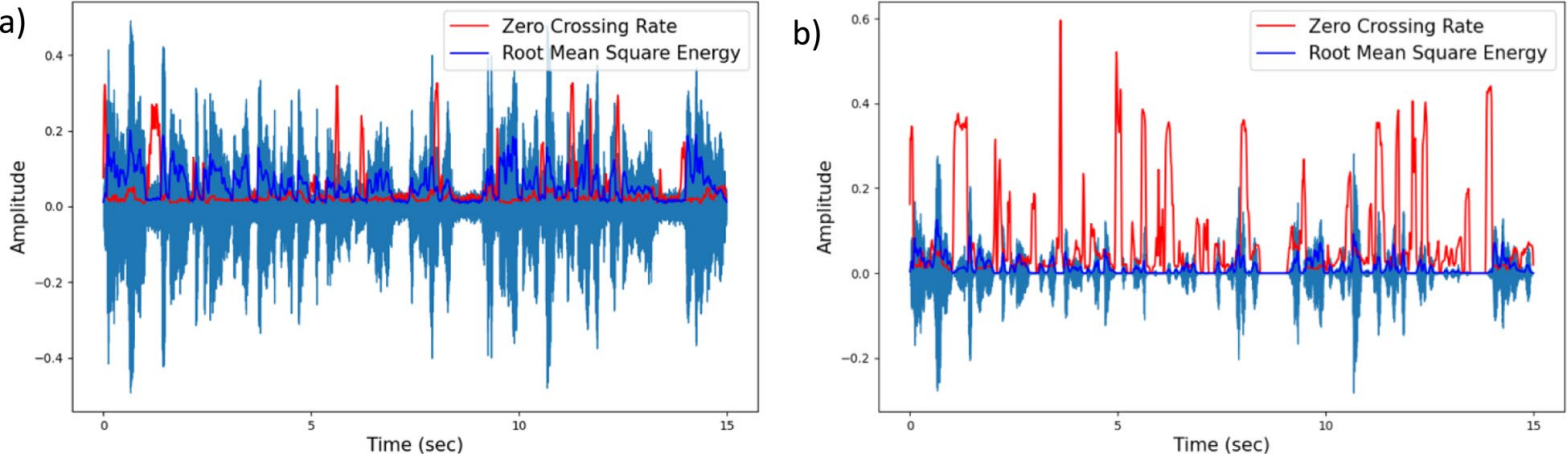
Time series plots for the same utterance from a sample AWS participant. **a)** Ambient noise is retained in this signal. Regions with no vocal activity have ZCR values in the similar range as regions with vocal activity. **b)** Ambient noise is removed from signal using spectral gating noise reduction. Non-vocal regions have a zero ZCR value while vocal regions have higher ZCR values

In other words, ZCR counts the number of times the signal changes sign, going from positive to negative or from negative to positive. The ZCR value is a representation of the silent and non-silent activity in the signal. In a signal that has the background noise retained, any region with no vocal activity shows higher ZCR values than any other region in the audio clip (Fig. 1b). However, this might not be the case when the ambient noise is very high (Fig. 2a). The silent regions with high ambient noise would not be detected as noise-only regions and would fail to have a comparatively higher ZCR in the audio file. On the other hand, for regions with complete silence (e.g., in a silent region of a noise-reduced file), the ZCR is expected to be zero (Fig. 2b). Thus, to be able to use data with high ambient noise, it is useful to perform noise reduction and detect the silent regions (i.e., without vocal activity) by checking for regions with ZCR values equal to zero.

### ATAS algorithm

**Fig. 3:**
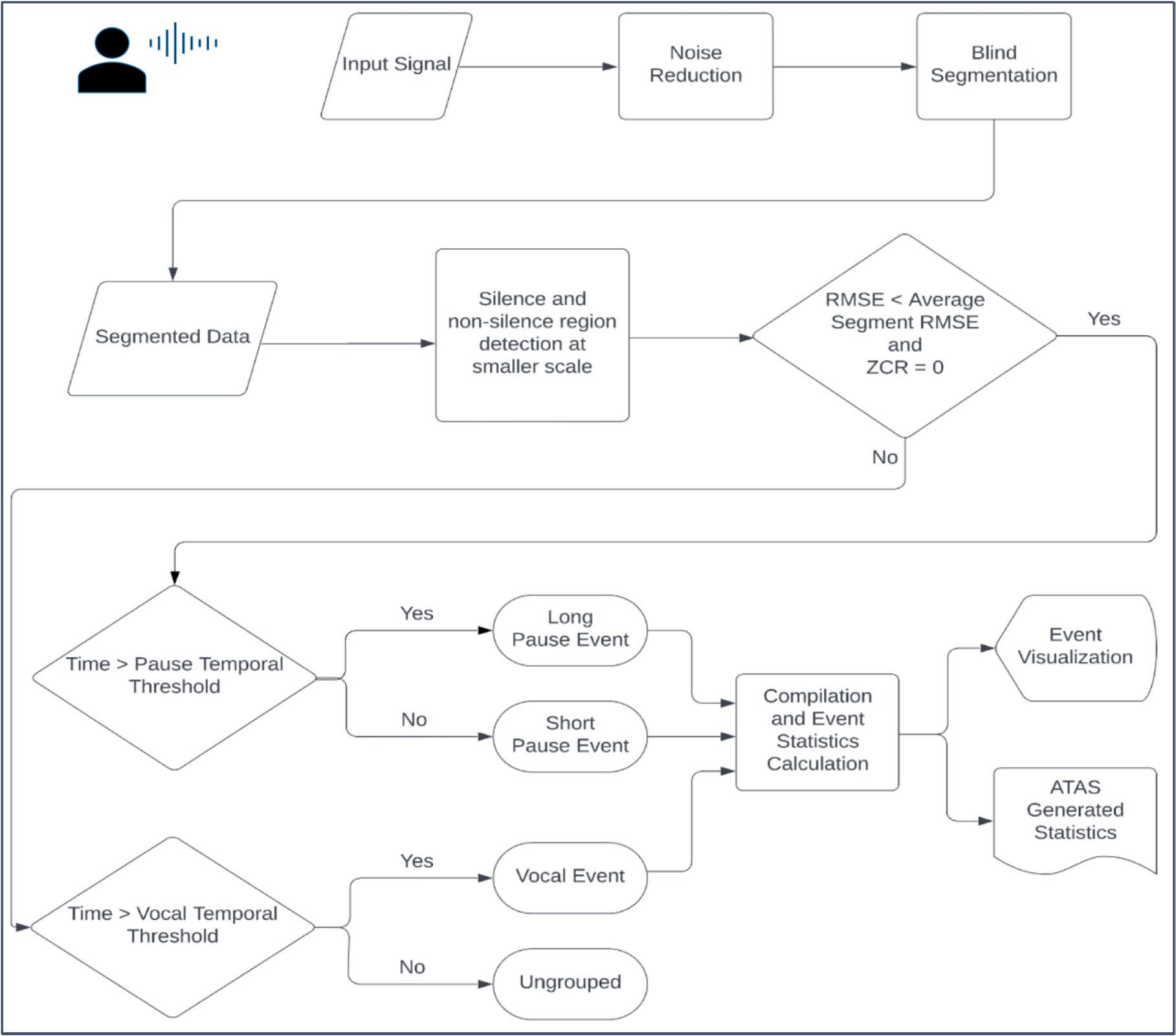
Flowchart depicting the various steps in ATAS

**Fig. 4:**
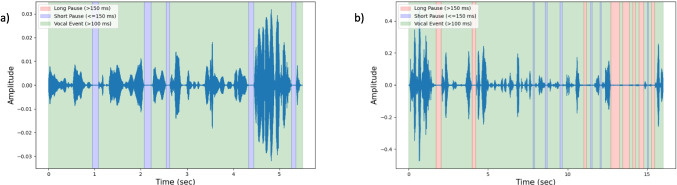
Pause and vocal event detection in time series plot of oral reading of “So here we are in Friuli, tucked away in a remote corner of the Alpine foothills in northeastern Italy, at a little restaurant,” by sample AWNS **a)** and AWS **b)** participants

The calculation of the amplitude threshold for each acoustic signal was a multi-step process. First, the normalized signal was blindly segmented into non-overlapping 3-s clips. Each of these segments was further analyzed separately. A short time analysis for a window size of 50 ms with step size of 1 ms was used to obtain short time RMSE and the ZCR values. The RMSE values were compared to the mean RMSE of the 3-s segment and the ZCR values were checked to be zero or non-zero, which were both features that were used to determine the thresholds. The regions of the signal with RMSE values greater than the mean RMSE and a non-zero ZCR value were classified as non-silent events, while the regions with RMSE values less than the mean RMSE and ZCR values equal to zero were classified as silent events. Further classification of the silent and non-silent regions into vocal and pause events was based on another set of thresholds: temporal – vocal and pause thresholds. Vocal and pause thresholds were predefined and set to be 100 ms and 50 ms, respectively. Thus, only silent regions greater than or equal to 50 ms were classified as pause events and non-silent regions greater than 100 ms were classified as vocal events. The pause events were further classified into short (50–150 ms) and long (>150 ms) pauses. Multiple speech and pause event outputs were derived from the approach used for the ATAS (Fig. 3), which were described as the ATAS measures. In Fig. 4, vocal pause events detected using ATAS are visualized as a time series plot that is coded for the different events detected.

### ATAS measurements and speech sample classification

Seven ATAS metrics (speech rate, total pause time, pause count, mean pause duration, mean vocal duration, pause duration variability, vocal duration variability) were calculated for pause and vocal events. In a follow-up exploratory analysis, ATAS metrics were separately calculated for short and long pause events. We conducted a multicollinearity check using the variance inflation factor (VIF), which indicated no severe multicollinearity (VIF < 10) among predictors. We then performed a statistical summary of continuous variables, assessing normality through Shapiro–Wilk tests (Shapiro & Wilk, 1965). Some variables, such as pause time, mean pause duration, and long pause duration variability, exhibited non-normal distributions, prompting log transformations. Log transformation improved normality for mean pause duration but was less effective for pause time and long pause duration variability. A homoscedasticity check was also performed using the Breusch–Pagan test (Breusch & Pagan, 1979), which detected heteroscedasticity in speech rate, pause time, and long pause duration variability, suggesting the need for weighted regression.

Additionally, we assessed overdispersion in Poisson models and found that pause count, long pause count and short pause count exhibited overdispersion, warranting the use of negative binomial models. To analyze continuous variables, we applied generalized linear models (GLM), using a weighted gamma GLM for pause time and long pause duration and a weighted Gaussian GLM for speech rate; while Gaussian GLMs were used for other continuous metrics. For count data, we fitted a standard Negative Binomial model to address overdispersion (α > 0). Group statistics and visualizations were used to illustrate group differences. Statistical significance was determined at α =.05. Our results included estimated coefficients, standard errors, and significance levels, providing insights into the effects of predictors (group, sex, and age) on speech and pause-related ATAS metrics. Subsequently, we applied the Benjamini–Hochberg procedure to control for the false discovery rate (FDR) amid multiple testing (Benjamini & Hochberg, 1995). This was implemented with a designated FDR threshold of .1, in order to mitigate the likelihood of type I errors given the multiple comparisons conducted.

We checked if the ATAS-generated features were useful in the automatic classification of the speech samples as AWNS speech samples and AWS speech samples using machine learning classifiers; 80% of the speech samples were used for training the models, while the remaining 20% were used for testing them. Classifiers such as decision trees and random forest based on ATAS metrics were tested (Breiman, 2001; Quinlan, 1986). The decision tree model was trained using all 13 ATAS metric features, including those based on long and short pauses, while the random forest model was trained using a subset of seven features selected for their statistical significance. The models were checked for validity and stability. Repetitive random sampling was used to generate multiple training and testing data sets and to select optimal hyper-parameters for the model. The entire dataset was then trained using the selected hyper-parameters. It was then validated using a 3-fold leave-one-out cross-validation process to estimate how well a model would generalize to new, unseen data.

We used the time-series data of the vocal and pause events of each audio file obtained from the ATAS pipeline along with the ATAS metrics in a long short-term memory model (Hochreiter & Schmidhuber, 1997). The data consisting of 35 audio files was split into training, testing, and validation groups as 29, four, and two files, respectively, without any overlap between the segments. Each segment had 20 events and nine features each. These features included the same set used for the random forest model, along with two additional continuous metrics: event type and event duration. The data were normalized, and the training data were augmented using data rotation. This was then provided as input to the long short-term memory model in order to train it as a classifier to distinguish speech in AWS and AWNS.

## Results

We found a considerable number of ATAS metrics to differ significantly between AWS and AWNS, and a lesser number of ATAS metrics satisfied our adjusted Benjamini–Hochberg threshold, revealing that AWS generally exhibit more pausing in speech. Mean values and other descriptive statistics for each of the 13 metrics are provided in Table 1. Inferential group statistics for 13 metrics are provided in Table 2 and Table 3. The predictors group and sex in Tables 2 and 3 represent categorical comparisons, with group reflecting the difference between the AWS group and the reference group (AWNS) and sex representing the difference between males and the reference category (females).

**Table 1:** Descriptive statistics for AWNS and AWS groups

Metric	Group	Mean	SD	Max	Min
Speech rate (words per minute)	AWNS	163.12	23.43	201.91	120.05
	AWS	127.60	38.11	180.85	53.55
Total pause time (s)	AWNS	16.48	4.68	24.47	10.55
	AWS	30.68	22.40	76.88	10.08
Pause count	AWNS	58.00	12.97	80	35
	AWS	86.47	56.37	250	35
Mean pause duration (ms)	AWNS	286.51	62.15	429.14	197.40
	AWS	338.14	84.40	519.09	245.80
Mean vocal duration (ms)	AWNS	1258.14	226.64	1758.19	863.55
	AWS	1250.82	358.11	1966.42	751.38
Pause duration variability	AWNS	.89	.11	1.09	.71
	AWS	.90	.16	1.23	.66
Vocal duration variability	AWNS	.74	.07	.86	.63
	AWS	.82	.10	.98	.61
Mean long pause duration (ms)	AWNS	450.99	75.42	620.46	330.48
	AWS	484.02	108.23	701.18	359.22
Mean short pause duration (ms)	AWNS	86.01	5.36	94.00	76.00
	AWS	90.41	6.57	99.68	78.29
Long pause duration variability	AWNS	.53	.08	.72	.41
	AWS	.61	.18	.96	.40
Short pause duration variability	AWNS	.33	.03	.39	.27
	AWS	.31	.04	.36	.23
Long pause count	AWNS	31.11	7.23	46	21
	AWS	55.12	38.54	168	22
Short pause count	AWNS	26.89	8.74	47	14
	AWS	31.35	18.35	82	13

**Table 2:** ATAS-based inferential statistics

Metric	Model	Predictor	*B*	SE	*Z*	*p*
Speech rate	Weighted GLM Gaussian	Intercept	166.30	17.10	9.72	<.001***
		Group	– 34.81	11.56	– 3.01	.003**
		Sex	4.61	11.47	.40	.69
		Age	–.18	.46	–.40	.69
Total pause time (s)	Weighted gamma GLM	Intercept	2.68	.30	8.85	<.001***
		Group	.59	.21	2.87	.004**
		Sex	.13	.20	.63	.53
		Age	.002	.01	.20	.84
Pause count	Negative binomial	Intercept	3.95	.23	17.12	<.001***
		Group	.37	.15	2.50	.01*
		Sex	.16	.15	1.07	.28
		Age	.001	.01	.13	.90
	Dispersion (α)		.15	.04	3.97	<.001
Mean pause duration (ms)	GLM Gaussian	Intercept	5.66	.12	46.86	<.001***
		Group	.16	.08	2.01	.04*
		Sex	.05	.08	.62	.53
		Age	–.001	.003	–.45	.65
Mean vocal duration (ms)	GLM Gaussian	Intercept	1422.39	141.23	10.07	<.001**
		Group	37.21	95.46	.39	.70
		Sex	– 301.78	94.73	– 3.19	.001**
		Age	.11	3.77	.03	.98
Pause duration variability	GLM Gaussian	Intercept	.83	.08	11.05	<.001***
		Group	–.004	.05	–.08	.94
		Sex	–.01	.05	–.23	.82
		Age	.002	.002	1.10	.27
Vocal duration variability	GLM Gaussian	Intercept	.75	.04	17.58	<.001***
		Group	.09	.03	3.09	.002**
		Sex	–.06	.03	– 1.96	.05
		Age	.001	.001	.45	.65

* = p <.05; ** = p <.01; *** = p < 0.001

**Table 3:** ATAS group statistics for long and short pauses

Metric	Model	Predictor	*b*	SE	*z*	*p*
Mean long pause duration (ms)	GLM Gaussian	Intercept	440.39	50.10	8.79	<.001***
		Group	30.00	33.86	.89	.38
		Sex	30.33	33.60	.90	.37
		Age	–.20	1.34	–.15	.88
Mean short pause duration (ms)	GLM Gaussian	Intercept	87.88	3.25	27.07	<.001***
		Group	4.87	2.19	2.22	.03*
		Sex	–.94	2.18	–.43	.67
		Age	–.04	.09	–.49	.62
Long pauses duration variability	Weighted gamma GLM	Intercept	–.68	.12	– 5.52	<.001***
		Group	.13	.08	1.56	.12
		Sex	–.06	.08	–.71	.48
		Age	.002	.003	.75	.45
Short pauses duration variability	GLM Gaussian	Intercept	.35	.02	19.61	<.001***
		Group	–.01	.01	–.52	.60
		Sex	–.01	.01	–.46	.64
		Age	–.001	<.001	– 1.52	.13
Long pause count	Negative binomial	Intercept	3.38	.25	13.41	<.001***
		Group	.55	.16	3.41	<.001***
		Sex	.14	.16	0.87	.39
		Age	–.001	.01	–.0.09	.93
	Dispersion (α)		.18	.05	3.86	<.001
Short pause count	Negative Binomial	Intercept	3.11	.23	13.56	<.001***
		Group	.11	.15	0.76	.45
		Sex	.17	.15	1.17	.24
		Age	.003	.01	.42	.67
	Dispersion (α)		.13	.04	3.43	.001

*= *p* <.05; ** = *p* <.01; *** = *p* <.001

Speech rate was significantly lower in the AWS group compared to controls. However, sex and age did not have a meaningful impact on speech rate. AWS participants exhibited significantly longer total pause durations than AWNS, while sex and age were not significant predictors. Similarly, the AWS group had a higher number of pauses overall, while age and sex did not significantly influence pause count. In terms of mean pause duration, AWS participants had significantly longer pauses on average compared to controls, whereas sex and age did not play a significant role.

**Fig. 5:**
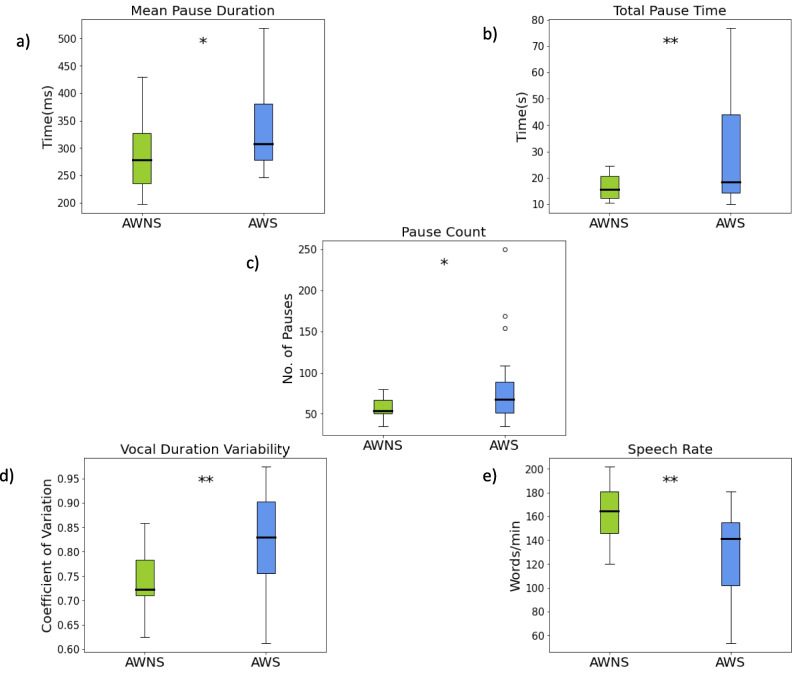
Differences in ATAS metrics between AWS and AWNS for pause and vocal events. **a)** Mean pause duration in milliseconds (ms). **b)** Total pause time in seconds (s). **c)** Pause count. **d)** Vocal duration variability or coefficient of variability. **e)** Speech rate in words per minute (WPM). * = *p* <.05; ** = *p* <.01; *** = *p* <.001

**Fig. 6:**
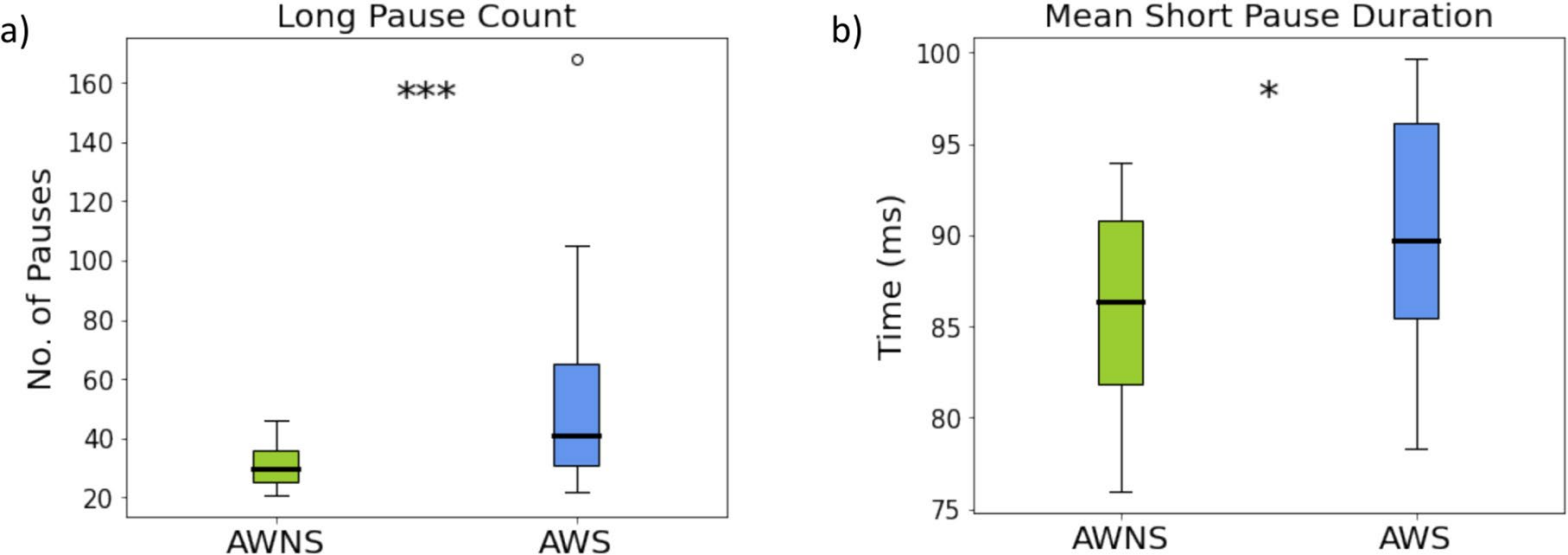
Bar plots depicting the differences between AWS and AWNS for long and short pause event metrics generated by ATAS. **a)** Long pause count. **b)** Mean short pause duration in milliseconds (ms). * = *p* <.05; *** = *p* <.001

Mean vocal duration did not differ between AWS and control groups, but male participants exhibited significantly shorter vocal durations than females. Age had no effect on vocal duration. Pause duration variability remained consistent across groups and sexes, with no significant influence from age. However, vocal duration variability was significantly greater in AWS participants compared to controls. The effect of sex was marginal, while age did not predict vocal duration variability. While significant differences were found, most dependent variables displayed substantial variability across groups, indicating some overlap (see Figs. 5 and 6).

Mean duration of long pauses did not differ significantly between AWS and AWNS groups, nor were there notable differences based on sex or age. However, the mean duration of short pauses was significantly higher in AWS compared to AWNS, while sex and age were not significant predictors. Variability in long and short pause durations showed no significant differences across groups, sexes, or age ranges. However, the AWS group produced a significantly greater number of long pauses compared to AWNS participants, while age and sex had no significant effect. For short pause count, there was no significant difference between AWS and AWNS groups, and age and sex were also not significant predictors.

**Fig. 7:**
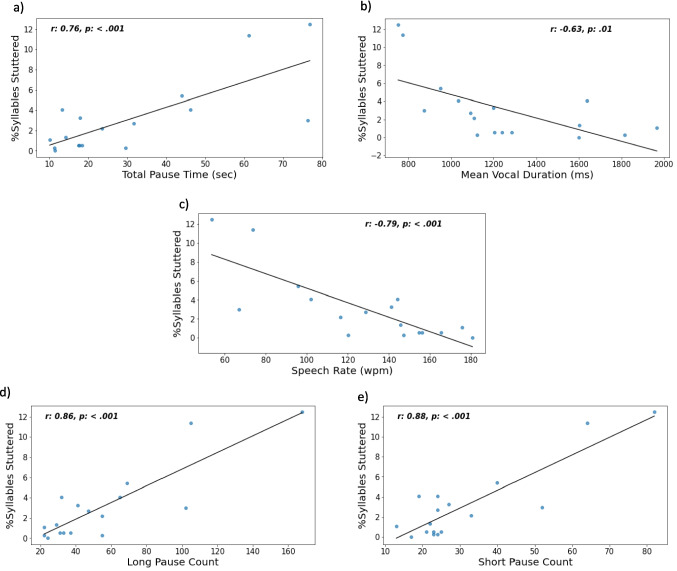
Correlations between percent syllables stuttered (%SS) and ATAS metrics: **a)** total pause time (s); **b)** mean vocal duration (ms); **c)** speech rate (words per minute); **d)** long pause count; and **e)** short pause count

**Fig. 8:**
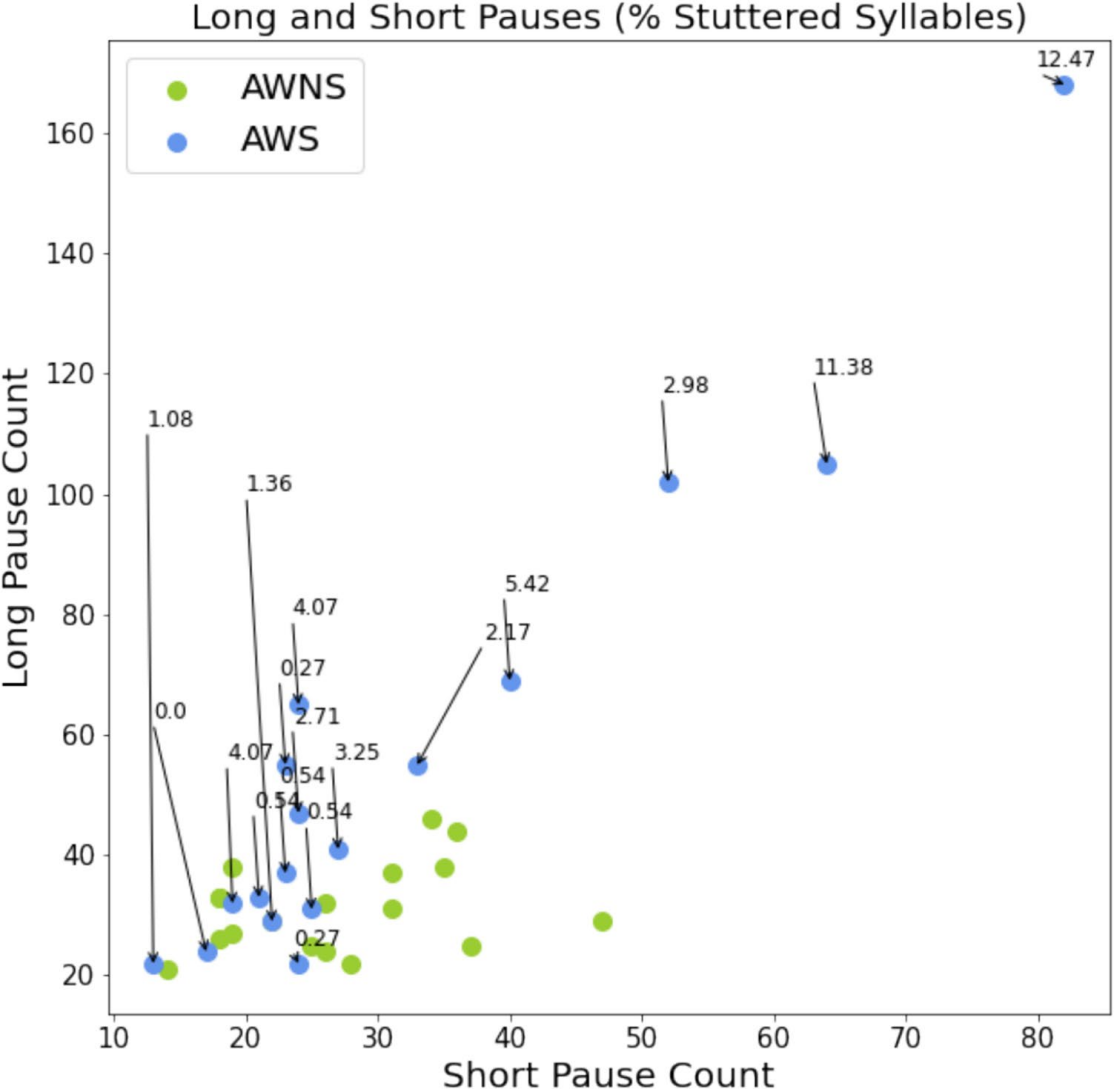
The relation between long pause count, short pause count and percent syllables stuttered (%SS). AWNS data points are *green*. AWS data points (*blue*) are labeled with %SS

To determine the relationship between our ATAS metrics and stuttering frequency, as measured by %SS, series of correlations were calculated. Speech rate decreased as %SS increased, (*r* = –.79, *p* = <.001). Total pause time increased with %SS, (*r* =.76, *p* = <.001). Pause count was also correlated with %SS. As shown in Fig. 7, we observed a strong correlation between long pause count and %SS (*r* =.86, *p* <.001) and short pause count and %SS (*r* =.88, *p* <.001). Mean vocal duration, but not pause duration, was negatively correlated with %SS (*r* = –.63, *p* =.01). The other ATAS metrics were not significantly correlated with %SS. To highlight potential individual differences among AWS regarding the association between pause time and stuttering frequency, we visualized %SS across individual short and long pause count data points (See Fig. 8). Regarding long pauses (i.e., > 150 ms), a significant number of AWS participants produced more than 50 long pauses, while none of the AWNS participants did so. Not surprisingly, long pause count was strongly correlated with %SS. Regarding short pause (50–150 ms) count, AWS were similar to AWNS.

**Fig. 9:**
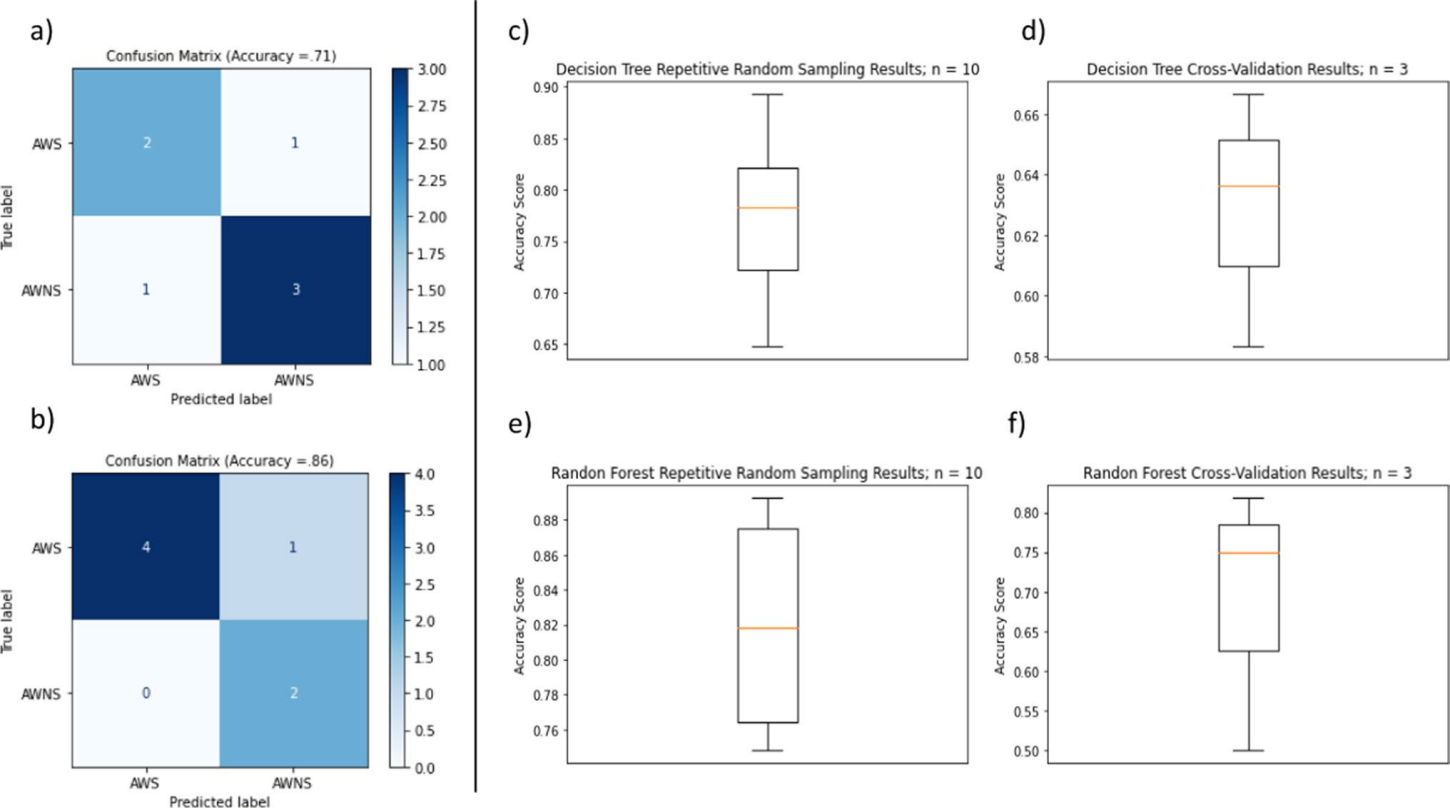
Results from exploratory classification modeling of AWS and AWNS based on pause and vocal event features calculated using ATAS. **a)** Confusion matrix for the automatic classification of speech as that of AWNS or AWS using the decision tree classifier. **b)** Confusion matrix for the automatic classification of speech as that of AWNS or AWS using the random forest classifier. **c)** Accuracy score from the 10-fold repetitive random sampling using the decision tree classifier. **d)** Accuracy score from the 3-fold cross validation using the decision tree classifier. **e)** Accuracy score from the 10-fold repetitive random sampling using the random forest classifier. f) Accuracy score from the 3-fold cross validation using the random forest classifier

**Fig. 10:**
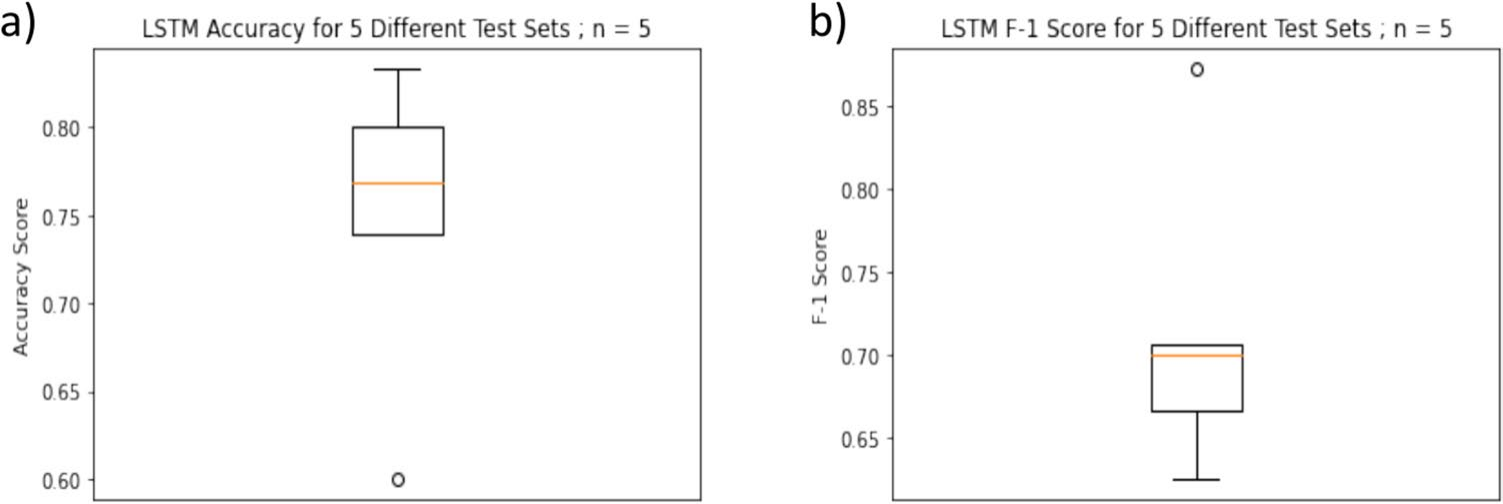
Results from exploratory classification modeling of the speech of AWS and AWNS based on the ATAS-generated time-series data and ATAS metrics. **a)** Accuracy scores from five different train-test splits using the long short-term memory model. **b)** F1 scores from five different train-test splits using the long short-term memory model

We next determined how accurate our ATAS metrics were in identifying group classification of AWS versus AWNS. Testing the vocal and pause ATAS metrics gave an accuracy of 71.43% for a decision tree (Fig. 9a). Selected features that were used for testing a random forest classifier generated an accuracy of 85.71% (Fig. 9b). Accuracy scores for the cross-validation and repetitive random sampling for both the types of classifiers show that in almost all the cases the scores are higher than 50% (Fig. 9c, d, e, and f). Accuracy scores for the cross-validation for the LSTM classifier also show that the scores are higher than random scores. F-1 scores for the LSTM classifier were higher than .60 (Fig. 10). While these results are based on specific training and testing splits and some alternative combinations might yield slightly lower performance, all three models generally demonstrated accuracies and F-1 scores that were higher than random chance.

## Discussion

The motivation for this study stems from the need for an objective, efficient, and automated method to assess speech fluency, particularly in individuals who stutter. To address this challenge, the study introduced ATAS, a computational tool that quantifies speech fluency by analyzing pauses and vocal events. Our first hypothesis was largely confirmed – AWS exhibited more pausing or hesitancy in speech compared to AWNS, as evidenced by slower speech rate, pause count, greater total pause time, and longer mean duration of pause events. The length of mean vocal duration did not differ between the groups. These findings suggest that slower speech in AWS may not be due to slower articulatory rate, given the duration of vocal events did not differ. Instead, it appears that AWS pause for relatively longer periods during speech. In our exploratory analyses of short and long pauses, AWS produced a greater number of long pauses but also longer short pauses compared to AWNS. In other words, the oral reading of AWS is particularly prone to the appearance of pauses or hesitations of considerable length, and this tendency is a fairly accurate marker of stuttering frequency. This increased pausing may be the key contributor to a slower speech rate, or more specifically, reading rate, a phenomenon that has been repeatedly previously observed in AWS (Prosek et al., 1979). Our findings also support previous arguments that a focus on pausing or hesitations in speech, regardless of categorization or classification of disfluency type, can provide a distinguishing feature of stuttering frequency (thus contributing to the more holistic concept of stuttering severity) (Prosek et al., 1979).

It should be stressed that our ATAS metrics were computed during a continuous speech that was highly controlled for linguistic and motor factors (i.e., oral reading). However, ATAS does not reveal underlying causes of the observed slower speech rate, greater pause time, higher pause count, and longer pause durations that characterize the speech of AWS. In this preliminary study, our ATAS metrics cannot map directly to overt stuttering behavior. It remains unclear if these ATAS metrics are the result of increased instances of overt stuttering behavior or subclinical difficulties in linguistic and speech-motor performance associated with categorical stuttering disfluency. However, our correlations revealed particularly strong relationships between stuttering frequency (i.e., %SS), speech rate, and total pause time. Those AWS participants with more instances of overt stuttering behavior while reading the passage also tended to have a slower speech rate and paused more than their AWS peers who stuttered less. Interestingly, the more frequent instances of overt stuttering behavior occurred, the shorter the duration of vocal events happened to be.

These findings suggest that overt stuttering behavior has a considerable impact on the efficient forward flow of speech – which we would refer to as ‘fluency.’ This argument may seem obvious, yet as shown in Fig. 7, this is not the case for all AWS participants. ATAS metrics of some AWS participants were relatively high, even compared to other AWS, despite low levels of perceived stuttering disfluency. This phenomenon leads to a larger question regarding the concept of fluency. Is %SS a valid measure of the level of speech ‘disfluency’? The answer may be affirmative if overt stuttering behavior is highly weighted in our subjective judgment (Yairi & Ambrose, 2004). The answer may be negative if fluency is quantified more holistically as the rhythmic and efficient forward flow of speech.

Our second central hypothesis was also largely confirmed – the speech of AWS was more temporally irregular than that of AWNS, as evidenced by greater variability in the duration of vocal events. This corresponds with a recent finding from our lab that AWS also exhibit reduced speech rhythm when reading the same passage, as measured by durational metrics (Boecher et al., 2023). Vocal duration variability likely measures the durational consistency of vocalic intervals, such as measures of speech rhythm like the pairwise variability index (PVI) (Grabe et al., 2002). Surprisingly, the duration of pause events was not relatively more variable in AWS. We also expected that these measures of temporal irregularity should be correlated with stuttering frequency, as indexed by %SS. Although the durational variability of vocal events was greater in AWS compared to AWNS, its association with %SS was not statistically significant.

Overall, it should be noted that the mapping between the elicitation of overt stuttering behavior and our ATAS metrics remains unclear. The strong negative relationship between mean vocal duration and %SS is suggestive that ATAS metrics are attuned to the habitual and transient interruptions in speech fluency that characterize overt stuttering behavior. Yet, ambiguity remains. Pausing output measures might reflect difficulty in the timing and sequencing of speech motor commands, including atypical respiratory function, which has been observed in AWS (Denny & Smith, 2000). It is possible that for some AWS, mean vocal duration could increase if their speech is marked by frequent prolongations. Pause count, as generated by ATAS, is nearly equal to the number of vocal events and could potentially be reduced in some AWS by a preponderance of repetitions. Some types of overt stuttering behavior, such as part-word or single-syllable-whole-word repetitions, may decrease the variability of vocal and pause events in some AWS, while increasing this variability in others. Temporal metrics of speech, which ATAS computes, are macro-level behavioral variables that likely emerge from any multitude of interacting factors – cognitive, linguistic, motoric, and pragmatic.

Our exploratory analysis using separate analyses for short and long pauses revealed the particular importance of long pauses as a distinguishing marker of stuttering. Even when the %SS score was relatively low for a self-identified AWS participant, the number of these long pauses was characteristically high compared to the number that AWNS produced. For example, one AWS participant with a %SS = 2.98% (which is below the traditional criteria of 3% for stuttering) still produced approximately 100 long pauses when reading our passage. Further analysis of the temporal patterns of pauses concerning the corresponding words in the passage could provide insights into the strategic long and short pauses by the speaker. AWS and AWNS might have certain differences in the way the short and long pauses are used when reading a particular sentence or uttering a certain uncommon word (e.g., “Friuli”) from the passage.

Our third hypothesis that ATAS metrics would accurately predict the group status of AWS or AWNS based on our reading sample was also confirmed. We constructed a classification model based on ATAS-generated metrics and time-series feature data and conducted an analysis based only on features calculated using ATAS metrics to train classifiers to blindly distinguish the speech of AWS from AWNS. When using ATAS metrics observed to be significantly different between AWS and AWNS, a random forest classifier was over 85% accurate in classifying the speech as that of an AWS or AWNS. The LSTM classifier based on ATAS-generated time-series data along with the ATAS metrics, classified the speech as that of an AWS and AWNS with an average accuracy of 75% and an F-1 scores higher than .60. This is suggestive that the temporal analysis of pause and vocal events provide added value to the assessment of stuttering.

### Clinical implications and future directions

As stated above, a significant number of SLPs have reported considerable uncertainty in their subjective judgment of overt stuttering behavior, such as stuttering frequency (Brundage et al., 2006). SLPs often rely on a variety of different assessment methodologies, such as the type of instrument or the unit of measurement, which can impact the accuracy and reliability of judgment of stuttering severity (Davidow & Scott, 2017; Valente et al., 2015). The accuracy of judgment may also depend on the amount of clinical experience of the SLP (Brundage et al., 2006; Bainbridge et al., 2015). Multiple studies based on the frequency counts of stuttering have reported the intra-judge agreement between raters to be low (Curlee, 1981; Martin & Haroldson, 1981). The intricacies and overlap of the signs and symptoms of disfluent speech, along with the largely subjective nature of quantifying such speech, have contributed to false-positive errors in inferring the presence of stuttering (Byrd, 2018). Moreover, it has been shown that assessment by SLPs can be less reliable when multiple spoken measures are collected simultaneously, e.g., stuttered syllables in isolation and total syllables spoken, thus impacting the quality of the assessment (Davidow et al., 2023). Additionally, this can also lead to low agreement among SLPs.

Given the complexities and ambiguities associated with the assessment and diagnosis of stuttering, the ability of the SLP to conduct a quick and objective temporal analysis of speech is likely to be additive to the best current clinical practice. The use of ATAS may be particularly helpful in the assessment of stuttering in children with unique behavioral profiles. In other words, future work is necessary to determine if ATAS is useful in distinguishing disfluent speech across different populations ranging from cluttering, autism spectrum, bilingualism, and language impairment. The inclusion of automated methods in the clinical assessment of stuttering would aid in providing more consistency along with simultaneous calculation of multiple vocal and pause event-related statistical features. It is important to have a consistent assessment of overt stuttering in order to increase the reliability of the assessment by SLPs. Making the assessment based on standardized parameters such as temporal thresholds will add to the consistency of the method. The use of assessment tools such as the SSI-4 involves the use of standard reading passages, including the “Friuli” passage used in this current study. The use of these or similar standardized passages for the acoustic analysis in terms of the pause and vocal events will prove to be very convenient for SLPs. Moreover, the ability of ATAS to visualize audio data in the form of distinct pause and vocal events may provide more confidence in assessment.

## Limitations

In the course of this preliminary study, we have identified a number of limitations that could be addressed in future studies. The first limitation is that our speaking task was limited to oral reading. This was purposeful due to the desire to control factors of speech and language that come with spontaneous and conversational speech. Future analyses of spontaneous speech using ATAS would be helpful to gain a better understanding of the temporal nature of young children who stutter, who are close to mean age of onset, yet are too young to provide reliable oral reading*.* A further limitation is related to the varied nature of pausing. As stated in the Introduction, ATAS characterizes all silent (non-vocal) intervals greater than 50 ms as “pause events.” However, the concept of the speech pause is multifunctional, particularly in relation to stuttering. Pausing in speech may convey difficulty in various speech-language processes. Pause characteristics are also related to respiratory dynamics and the tendency to self-repair or correct speech-language production, e.g., interjection, false start (Duez, 1982). Pauses can also be acoustically classified into three major types: silent, breath, and filled pauses (Igras-Cybulska et al., 2016). Filled pauses are pseudo-words or vocal events that do not impact the meaning of the sentence (Swerts, 1998). Thus, ATAS parameters would classify a “filled pause” as a vocal event. Breath pauses are typically only recorded if the signal-to-noise ratio is high or if the recording microphone is very close to the speaker (Igras-Cybulska et al., 2016). In such an event, a breath pause could also be identified by ATAS as a vocal event. Pauses may also be used as a strategy to prevent or ameliorate moments of stuttering (Reitzes, 2006). On the contrary, pausing or hesitations may be a sign of stuttering behavior – particularly in the form of speech blocks and repetitions. Short pauses, for example, often occur in repeated utterances, such as part-word repetitions (Au-Yeung & Howell, 1998; Goldman-Eisler, 1972).

## Conclusion

In this study, we developed a methodology for ATAS to detect vocal and pause events and generate statistical features corresponding to these events in the speech of AWS and AWNS. Overall, AWS exhibited more pausing or hesitancy in speech compared to AWNS, as evidenced by slower speech rate, greater total pause time, and longer mean duration of pause events. Of particular importance in distinguishing the speech of AWS and AWNS is the appearance of pauses or hesitations greater than 150 ms. Numerous pause and vocal metrics acquired from ATAS were correlated with stuttering frequency, which is suggestive that automatically detected temporal metrics of pause and vocal events within continuous speech are highly associated with stuttering. An attempt to automatically distinguish the speech of AWS and AWNS using machine learning classifiers resulted in higher than random accuracy in most of the cases. The accuracy of the models is influenced by the specific training and testing data splits used. The results are limited by the size and diversity of the data, suggesting that larger training datasets could improve model performance and reliability. It is important to consider training the machine learning classifiers on a bigger dataset to generalize the models and improve the vocal and pause event-based learning. Our findings support the potential of ATAS as an objective and efficient method for the assessment of overt stuttering behavior that can be additive and complementary to previously applied diagnostic and assessment methods.

## Data Availability

All the data for adults who stutter can be accessed through the FluencyBank database: https://sla.talkbank.org/TBB/fluency/Voices-AWS/readings

## Appendix 1. Reading passage taken from the Stuttering Severity Instrument-Fourth Edition (Riley & Bakker, 2009)

So here we are in Friuli, tucked away in a remote corner of the Alpine foothills in northeastern Italy, at a little restaurant. I have to admit that when I travel, history is not the first thing on my mind. Food and wine are. And that’s what sold me on Friuli. It is famous as a source of some of Italy’s best white wines. We went primarily in search of wines, unaware that we would soon make a culinary detour. Occupying the extreme northeast corner of Italy, Friuli’s scenery ranges from rugged coastline along the eastern border to placid plains in the West and the majestic Alps in the North, where Italy butts up against Austria. Directly to the South is Venice, just a little more than an hour and a half away. Though off the beaten tourist track, Friuli is hard in the path of history. Standing at one of the major crossroads between Western Europe and the East, it was conquered by just about everyone who passed by. As a result, things look different here. Rather than the familiar cultural overlay of most of Italy, the central European influence is readily apparent in Friuli. The architecture tends more toward Austrian grandeur than Tuscan simplicity. Here, you’ll find gray stone castles rather than sun-drenched villas. The people look different, too, taller and blonder than southern Italians and with plenty of German and Central European surnames.

## Code availability

Code for the analyses in this article is available at (https://github.com/vishrutay/ATAS).

## References

[CR1] Afroz, F., & Koolagudi, S. G. (2019). Recognition and classification of pauses in stuttered speech using acoustic features. *2019 6th International Conference on Signal Processing and Integrated Networks (SPIN)* (pp. 921–926). IEEE. 10.1109/SPIN.2019.8711569

[CR2] Arenas, R. M. (2017). Conceptualizing and investigating the contextual variability of stuttering: The speech and monitoring interaction (SAMI) framework. *Speech, Language and Hearing,**20*(1), 15–28. 10.1080/2050571X.2016.1221877

[CR3] Atmaja, B. T., & Akagi, M. (2020a). The effect of silence feature in dimensional speech emotion recognition. arXiv preprint arXiv:2003.01277. Retrieved November 28, 2023, from https://arxiv.org/abs/2003.01277. Accessed 28 Nov 2023.

[CR4] Atmaja, B. T., & Akagi, M. (2020b). Deep multilayer perceptrons for dimensional speech emotion recognition. *2020 Asia-Pacific Signal and Information Processing Association Annual Summit and Conference (APSIPA ASC)* (pp. 325–331). IEEE. https://ieeexplore.ieee.org/abstract/document/9306219. Accessed 5 Aug 2023.

[CR5] Au-Yeung, J., & Howell, P. (1998). Lexical and syntactic context and stuttering. *Clinical Linguistics & Phonetics,**12*(1), 67–78. 10.3109/02699209808985213

[CR6] Bainbridge, L. A., Stavros, C., Ebrahimian, M., Wang, Y., & Ingham, R. J. (2015). The efficacy of stuttering measurement training: Evaluating two training programs. *Journal of Speech, Language, and Hearing Research: JSLHR,**58*(2), 278–286. 10.1044/2015_JSLHR-S-14-0200 25629956 10.1044/2015_JSLHR-S-14-0200 PMC4675120

[CR7] Barrett, L., Hu, J., & Howell, P. (2022). Systematic review of machine learning approaches for detecting developmental stuttering. *IEEE/ACM Transactions on Audio, Speech, and Language Processing,**30*, 1160–1172. 10.1109/TASLP.2022.3155295

[CR8] Beltrame, J. M., Viera, R. A. T., Tamanaha, A. C., Arcuri, C. F., Osborn, E., Perissinoto, J., & Schiefer, A. M. (2011). Comparison of pausing behavior in children who stutter and children who have Asperger syndrome. *Journal of Fluency Disorders,**36*(4), 280–284. 10.1016/j.jfludis.2011.07.001 22133405 10.1016/j.jfludis.2011.07.001

[CR9] Benjamini, Y., & Hochberg, Y. (1995). Controlling the false discovery rate: A practical and powerful approach to multiple testing. *Journal of the Royal Statistical Society,**57*(1), 289–300. 10.1111/j.2517-6161.1995.tb02031.x

[CR10] Bloodstein, O., & Bernstein Ratner, N. (2007). *A Handbook on Stuttering* (6th ed.). Delmar Cengage Learning.

[CR11] Bloodstein, O., Ratner, N. B., & Brundage, S. B. (2021). *A Handbook on Stuttering* (7th ed.). Plural Publishing. https://play.google.com/store/books/details?id=Abw0EAAAQBAJ

[CR12] Boecher, J., Franich, K., Chow, H. M., & Usler, E. R. (2023). Dysrhythmic speech is a characteristic of developmental stuttering in adults: A quantitative analysis using duration- and interval-based rhythm metrics. *Journal of Speech, Language, and Hearing Research,**68*(4), 1618–1633. 10.31234/osf.io/zqyjn 10.1044/2024_JSLHR-24-00076 40080872

[CR13] Bothe, A. K. (2008). Identification of children’s stuttered and nonstuttered speech by highly experienced judges: Binary judgments and comparisons with disfluency-types definitions. *Journal of Speech, Language, and Hearing Research: JSLHR,**51*(4), 867–878. 10.1044/1092-4388(2008/063) 18658057 10.1044/1092-4388(2008/063)

[CR14] Breiman, L. (2001). Random forests. *Machine Learning,**45*(1), 5–32. 10.1023/A:1010933404324

[CR15] Breusch, T. S., & Pagan, A. R. (1979). A simple test for heteroscedasticity and random coefficient variation. *Econometrica: Journal of the Econometric Society,**47*(5), 1287. 10.2307/1911963

[CR16] Brundage, S. B., Bothe, A. K., Lengeling, A. N., & Evans, J. J. (2006). Comparing judgments of stuttering made by students, clinicians, and highly experienced judges. *Journal of Fluency Disorders,**31*(4), 271–283. 10.1016/j.jfludis.2006.07.002 16982086 10.1016/j.jfludis.2006.07.002

[CR17] Byrd, C. T. (2018). Assessing bilingual children: Are their disfluencies indicative of stuttering or the by-product of navigating two languages? *Seminars in Speech and Language,**39*(4), 324–332. 10.1055/s-0038-1667161 30142643 10.1055/s-0038-1667161

[CR18] Campione, E., & Véronis, J. (2002). A large-scale multilingual study of silent pause duration. In *Speech prosody* (Vol. 2002, pp. 199–202). Retrieved November 1, 2023, from 10.21437/speechprosody.2002-35. Accessed 1 Nov 2023.

[CR19] Curlee, R. F. (1981). Observer agreement on disfluency and stuttering. *Journal of Speech and Hearing Research,**24*(4), 595–600. 10.1044/jshr.2404.595 7035743 10.1044/jshr.2404.595

[CR20] Davidow, J. H., & Scott, K. A. (2017). Intrajudge and interjudge reliability of the stuttering severity instrument–fourth edition. *American Journal of Speech-Language Pathology / American Speech-Language-Hearing Association,**26*, 1105–1119. 10.1044/2017_AJSLP-16-0079 10.1044/2017_AJSLP-16-0079 28841724

[CR21] Davidow, J. H., Ye, J., & Edge, R. L. (2023). The reliability of simultaneous versus individual data collection during stuttering assessment. *International Journal of Language & Communication Disorders / Royal College of Speech & Language Therapists,**58*(4), 1251–1267. 10.1111/1460-6984.12860 10.1111/1460-6984.12860 36861494

[CR22] Denny, M., & Smith, A. (2000). Respiratory control in stuttering speakers. *Journal of Speech, Language, and Hearing Research: JSLHR,**43*(4), 1024–1037. 10.1044/jslhr.4304.1024 11386469 10.1044/jslhr.4304.1024

[CR23] Duez, D. (1982). Silent and non-silent pauses in three speech styles. *Language and Speech,**25*(1), 11–28. 10.1177/002383098202500102

[CR24] Goldman-Eisler, F. (1972). Pauses, clauses, sentences. *Language and Speech,**15*(2), 103–113. 10.1177/002383097201500201 4653677 10.1177/002383097201500201

[CR25] Grabe, E., et al. (2002). Durational variability in speech and the rhythm class hypothesis. *Papers in Laboratory Phonology,**7*(515–546), 1–16. http://wwwhomes.uni-bielefeld.de/~gibbon/AK-Phon/Rhythmus/Grabe/Grabe_Low-reformatted.pdf.

[CR26] Green, J. R., Beukelman, D. R., & Ball, L. J. (2004). Algorithmic estimation of pauses in extended speech samples of dysarthric and typical speech. *Journal of Medical Speech-Language Pathology,**12*(4), 149–154. https://www.ncbi.nlm.nih.gov/pmc/articles/PMC2902000/. 20628555 PMC2902000

[CR27] Hamre, C. (1992). Stuttering prevention I: Primacy of identification. *Journal of Fluency Disorders,**17*(1), 3–24. 10.1016/0094-730X(92)90017-K

[CR28] Hochreiter, S., & Schmidhuber, J. (1997). Long short-term memory. *Neural Computation,**9*(8), 1735–1780. 10.1162/neco.1997.9.8.1735 9377276 10.1162/neco.1997.9.8.1735

[CR29] Howell, P., Soukup-Ascencao, T., Davis, S., & Rusbridge, S. (2011). Comparison of alternative methods for obtaining severity scores of the speech of people who stutter. *Clinical Linguistics & Phonetics,**25*(5), 368–378. 10.3109/02699206.2010.538955 21434809 10.3109/02699206.2010.538955 PMC3314730

[CR30] Igras-Cybulska, M., Ziółko, B., Żelasko, P., & Witkowski, M. (2016). Structure of pauses in speech in the context of speaker verification and classification of speech type. *EURASIP Journal on Audio, Speech, and Music Processing,**2016*(1), 18. 10.1186/s13636-016-0096-7

[CR31] Inouye, J. M., Blemker, S. S., & Inouye, D. I. (2014). Towards undistorted and noise-free speech in an MRI scanner: Correlation subtraction followed by spectral noise gating. *The Journal of the Acoustical Society of America,**135*(3), 1019–1022. 10.1121/1.4864482 24606243 10.1121/1.4864482

[CR32] Johnson, W. (1961). *Stuttering and What you can do About it*. University of Minnesota Press. https://muse.jhu.edu/pub/23/monograph/book/32452

[CR33] Karimi, H., Jones, M., O’Brian, S., & Onslow, M. (2014). Clinician percent syllables stuttered, clinician severity ratings and speaker severity ratings: Are they interchangeable?: %SS and severity rating scales. *International Journal of Language & Communication Disorders,**49*(3), 364–368. 10.1111/1460-6984.12069 24304909 10.1111/1460-6984.12069

[CR34] König, A., Satt, A., Sorin, A., Hoory, R., Toledo-Ronen, O., Derreumaux, A., Manera, V., Verhey, F., Aalten, P., Robert, P. H., & David, R. (2015). Automatic speech analysis for the assessment of patients with predementia and Alzheimer’s disease. *Alzheimer’s & Dementia: The Journal of the Alzheimer’s Association,**1*(1), 112–124. 10.1016/j.dadm.2014.11.012 10.1016/j.dadm.2014.11.012 PMC4876915 27239498

[CR35] Krivokapić, J. (2009). Prosodic planning: Local and distant effects of phrase length on pause duration. *The Journal of the Acoustical Society of America,**125*(4 Supplement), 2573–2573. 10.1121/1.4783769

[CR36] Lea, C., Mitra, V., Joshi, A., Kajarekar, S., & Bigham, J. P. (2021). SEP-28k: A dataset for stuttering event detection from podcasts with people who stutter. *ICASSP 2021 - 2021 IEEE International Conference on Acoustics, Speech and Signal Processing (ICASSP)* (pp. 6798–6802). IEEE. 10.1109/ICASSP39728.2021.9413520

[CR37] Love, L. R., & Jeffress, L. A. (1971). Identification of brief pauses in the fluent speech of stutterers and nonstutterers. *Journal of Speech and Hearing Research,**14*(2), 229–240. 10.1044/jshr.1402.229 5558075 10.1044/jshr.1402.229

[CR39] Martin, R. R., & Haroldson, S. K. (1981). Stuttering identification: Standard definition and moment of stuttering. *Journal of Speech and Hearing Research,**24*(1), 59–63. https://www.ncbi.nlm.nih.gov/pubmed/7253630. 7253630

[CR40] Mcclay, H., & Osgood, C. E. (1959). Hesitation phenomena in spontaneous English speech. *Word,**15*, 19–44.

[CR41] Merriam-Webster. (n.d.). Merriam-Webster: America’s most trusted dictionary. Retrieved November 6, 2023, from https://www.merriam-webster.com/

[CR42] Neef, N. E., Anwander, A., Bütfering, C., Schmidt-Samoa, C., Friederici, A. D., Paulus, W., & Sommer, M. (2018). Structural connectivity of right frontal hyperactive areas scales with stuttering severity. *Brain: A Journal of Neurology,**141*(1), 191–204. 10.1093/brain/awx316 29228195 10.1093/brain/awx316 PMC5837552

[CR43] Orpella, J., Flick, G., Assaneo, M. F., Pylkkänen, L., Poeppel, D., & Jackson, E. S. (2023). *Reactive Inhibitory Control Precedes Overt Stuttering Events* (p. 2022.08.02.501857). bioRxiv. 10.1101/2022.08.02.501857 10.1162/nol_a_00138 PMC11192511 38911458

[CR44] Perkins, W. H. (1984). Stuttering as a categorical event. *The Journal of Speech and Hearing Disorders,**49*(4), 431–434. 10.1044/jshd.4904.431

[CR45] Prosek, R. A., Walden, B. E., Montgomery, A. A., & Schwartz, D. M. (1979). Some correlates of stuttering severity judgments. *Journal of Fluency Disorders,**4*(3), 215–222. 10.1016/0094-730X(79)90020-2

[CR46] Quinlan, J. R. (1986). Induction of decision trees. *Machine Learning,**1*(1), 81–106. 10.1007/BF00116251

[CR47] Ratcliff, A., Coughlin, S., & Lehman, M. (2002). Factors influencing ratings of speech naturalness in augmentative and alternative communication. *Augmentative and Alternative Communication,**18*(1), 11–19. 10.1080/aac.18.1.11.19

[CR48] Ratner, N. B., & MacWhinney, B. (2018). Fluency Bank: A new resource for fluency research and practice. *Journal of Fluency Disorders,**56*, 69–80. 10.1016/j.jfludis.2018.03.002 29723728 10.1016/j.jfludis.2018.03.002 PMC5986295

[CR49] Reitzes, P. (2006). Pausing: Reducing the frequency of stuttering. *The Journal of Stuttering Therapy, Advocacy, and Research,**1*, 64–78.

[CR50] Rieber, R. W., Breskin, S., & Jaffe, J. (1972). Pause time and phonation time in stuttering and cluttering. *Journal of Psycholinguistic Research,**1*(2), 149–154. 10.1007/BF01068104 24197576 10.1007/BF01068104

[CR51] Riley, G., & Bakker, K. (2009). *SSI-4: Stuttering severity instrument*. PRO-ED, An International Publisher.

[CR52] Shapiro, S. S., & Wilk, M. B. (1965). An analysis of variance test for normality (Complete Samples). *Biometrika,**52*(3/4), 591–611. 10.2307/2333709

[CR53] Sheikh, S. A., Sahidullah, Hirsch, F., & Ouni, S. (2022). Machine learning for stuttering identification: Review, challenges and future directions. *Neurocomputing,**514*, 385–402. 10.1016/j.neucom.2022.10.015

[CR54] Swerts, M. (1998). Filled pauses as markers of discourse structure. *Journal of Pragmatics,**30*(4), 485–496. 10.1016/S0378-2166(98)00014-9

[CR55] Tanchip, C., Guarin, D. L., McKinlay, S., Barnett, C., Kalra, S., Genge, A., Korngut, L., Green, J. R., Berry, J., Zinman, L., Yadollahi, A., Abrahao, A., & Yunusova, Y. (2022). Validating automatic diadochokinesis analysis methods across dysarthria severity and syllable task in amyotrophic lateral sclerosis. *Journal of Speech, Language, and Hearing Research: JSLHR,**65*(3), 940–953. 10.1044/2021_JSLHR-21-00503 35171700 10.1044/2021_JSLHR-21-00503 PMC9150739

[CR56] Teixeira, J. P., Fernandes, M. G., & Costa, R. A. (2017). Automatic determination of pauses in speech for classification of stuttering disorder. *Design, Development, and Integration of Reliable Electronic Healthcare Platforms* (pp. 132–149). IGI Global. 10.4018/978-1-5225-1724-5.ch008

[CR57] Tichenor, S., & Yaruss, J. S. (2018). A phenomenological analysis of the experience of stuttering. *American Journal of Speech-Language Pathology / American Speech-Language-Hearing Association,**27*(3S), 1180–1194. 10.1044/2018_AJSLP-ODC11-17-0192 10.1044/2018_AJSLP-ODC11-17-0192 30347062

[CR58] Toth, L., Hoffmann, I., Gosztolya, G., Vincze, V., Szatloczki, G., Banreti, Z., Pakaski, M., & Kalman, J. (2018). A speech recognition-based solution for the automatic detection of mild cognitive impairment from spontaneous speech. *Current Alzheimer Research,**15*(2), 130–138. 10.2174/1567205014666171121114930 29165085 10.2174/1567205014666171121114930 PMC5815089

[CR59] Valente, A. R. S., Jesus, L. M. T., Hall, A., & Leahy, M. (2015). Event- and interval-based measurement of stuttering: A review. *International Journal of Language & Communication Disorders / Royal College of Speech & Language Therapists,**50*(1), 14–30. 10.1111/1460-6984.12113 10.1111/1460-6984.12113 24919948

[CR60] Walsh, B., Bostian, A., Tichenor, S. E., Brown, B., & Weber, C. (2020). Disfluency characteristics of 4- and 5-year-old children who stutter and their relationship to stuttering persistence and recovery. *Journal of Speech, Language, and Hearing Research: JSLHR,**63*(8), 2555–2566. 10.1044/2020_JSLHR-19-00395 32692634 10.1044/2020_JSLHR-19-00395 PMC7872730

[CR61] Wingate, M. E. (1984). Pause loci in stuttered and normal speech. *Journal of Fluency Disorders,**9*(3), 227–235. 10.1016/0094-730X(84)90016-0

[CR62] Yairi, E., & Ambrose, N. G. (2004). Early Childhood Stuttering. *PRO-ED, Inc. *PRO-ED, Inc. http://www.proedinc.com

